# Upregulation of Nei-Like DNA Glycosylase 3 Predicts Poor Prognosis in Hepatocellular Carcinoma

**DOI:** 10.1155/2021/1301671

**Published:** 2021-10-08

**Authors:** Dongyu Wu, Guangcong Zhang, Jiamei Ma, Hongfen Wu, Ju Xiong, Xiaoxi Huang, Yuanyuan Tian, Taozhi Deng, Xiangyang Han, Xiaoning Sun, Tian Xiang, Xiangnan Yu, Xuemei Jiang

**Affiliations:** ^1^Department of Gastroenterology, Hainan General Hospital (Hainan Affiliated Hospital of Hainan Medical University), Haikou 570100, China; ^2^Department of Gastroenterology and Hepatology, Zhongshan Hospital of Fudan University, Shanghai 200030, China; ^3^Department of Gastroenterology, Central South University Xiangya School of Medicine Affiliated Haikou Hospital, Haikou 570100, China; ^4^Department of Gastroenterology, Sanya People's Hospital, Sanya 572000, China; ^5^Department of General Surgery, People's Hospital of Xinjiang Uygur Autonomous Region, Urumqi 830001, China; ^6^Hainan Medical University, Haikou 570100, China; ^7^Medical Examination, School of Tropical Medicine and Laboratory Medicine, Hainan Medical University, Haikou 570100, China

## Abstract

**Background:**

Accumulating evidence has suggested that Nei-like DNA glycosylase 3 (NEIL3) is associated with human tumors. However, there are few studies on the role of NEIL3 in hepatocellular carcinoma (HCC). The aim of this study was to investigate the expression profile of NEIL3 and its clinical relevance in HCC.

**Materials and Methods:**

A total of 130 HCC and corresponding nontumor tissues were collected to perform immunohistochemistry (IHC). The clinical relevance and prognostic value of NEIL3 in HCC were analyzed by the chi-square test, Kaplan–Meier analysis, the Cox proportional hazard model, and nomogram.

**Results:**

IHC showed that the NEIL3 protein level was remarkably upregulated in tumor tissues compared with nontumor tissues (fold change = 1.24; *P* < 0.001). High NEIL3 expression was significantly correlated with BCLC stage (*P*=0.004) and TNM stage (*P*=0.005). Overall survival (OS) and disease-free survival (DFS) rates in the high NEIL3 expression group were significantly worse than those in the low NEIL3 expression group (*P*=0.007 and *P*=0.004, respectively). Furthermore, subgroup analysis showed that high NEIL3 expression predicted worse OS and DFS for HCC patients with advanced TNM stage, poorly differentiated tumor, HBsAg positive, or cirrhosis. Multivariate analysis and the prognostic nomograms revealed that tumor NEIL3 level may serve as a promising prognostic indicator for OS and DFS in HCC patients.

**Conclusion:**

Our findings suggested that NEIL3 might be a potential prognosis assessment marker and therapeutic target for HCC patients.

## 1. Introduction

Hepatocellular carcinoma (HCC) is the dominant histological type of primary liver cancer and the third most common cause of cancer-related deaths worldwide [1]. Although early diagnostic modalities and treatment utilization have partially improved prognosis, the long-term clinical outcome of HCC patients remains poor due to recurrence and distant metastasis [2–5]. Therefore, novel prognostic factors for early-stage diagnosis and early recurrence are still urgently needed.

Nei-like DNA glycosylase 3 (NEIL3) is a member of the DNA glycosylase family homologous to the bacterial Fpg/Nei class [6]. A recent study showed that NEIL3 is implicated in protecting telomere integrity by repairing oxidative lesions of telomeric sequences in proliferating cells [7]. NEIL3 expression is restricted to proliferating cells [8, 9]. For instance, in adult humans, NEIL3 transcripts are only detectable in the thymus and testes [6, 9]. And in mice, NEIL3 has been detected in the thymus, bone marrow, spleen, and regenerative subregions of the brain [10–12]. Moreover, a recent study showed that NEIL3 expression is remarkably upregulated in 16 cancerous tissues [11]. Previous studies also have indicated that overexpression of NEIL3 is correlated with poor prognosis of astrocytoma and melanoma patients [13, 14]. Overall, these studies suggest that NEIL3 is important in actively proliferating cells.

To our knowledge, there are few studies investigating the role of NEIL3 in HCC. In Zhang's study, NEIL3 was found to be associated with the most frequent loss of heterozygosity using a high-throughput single nucleotide polymorphism (SNP) array, suggesting a relationship between NEIL3 genetic abnormalities and hepatocarcinogenesis [15]. However, the study did not further examine the relationship between NEIL3 expression and patient prognosis in clinical specimens. In the present study, to better understand the role of NEIL3 in HCC, we determined NEIL3 expression in 130 pairs of clinical specimens and hepatic cell lines. Moreover, we confirmed that NEIL3 overexpression is correlated with poor survival prognosis and could serve as an independent prognostic factor for HCC patients.

## 2. Materials and Methods

### 2.1. Patients and Tissue Specimens

A total of 130 HCC and corresponding nontumor tissues were collected from Hainan General Hospital (Haikou, China). All HCC patients were diagnosed based on pathological examination and underwent curative hepatectomy between February 2006 and August 2011. None of the patients received adjuvant therapy, such as radiotherapy or chemotherapy. Our follow-up data ended in April 2017. The survival time was calculated from the date of operation to death or the deadline for follow-up.

The main clinicopathological characteristics of the patients are summarized in Table 1. Briefly, there were 102 males and 28 females in the patient cohort, with a median age of 53.0 (range 19–89 years) at the time of surgery. There were 106 patients with liver cirrhosis and 24 without liver cirrhosis. Patients were classified according to the eighth edition of the tumor–node–metastasis (TNM) staging system and Barcelona clinic liver cancer (BCLC) staging system. Of the 130 patients, 94 tumors were in TNM stages I and II; 36 were in stages III and IV; 84 tumors were in BCLC stages 0–A; and 46 were in stages B and C.

### 2.2. Cell Culture

A human nontransformed hepatic cell line (L02) and seven human HCC cell lines (BEL-7402, SMCC-7721, Huh7, PLC/PRF/5, HepG2, Hep3B, SK-Hep-1, and HepG2) were used in this study. L02 and HepG2 were purchased from the Cell Bank of the Chinese Academy of Sciences (Shanghai, China). Cells were cultured in Dulbecco's modified Eagle's medium (Gibco, Carlsbad, CA, USA) supplemented with 10% fetal bovine serum (Gibco, Grand Island, NY, USA) and 1% penicillin/streptomycin (Corning, Lowell, MA, USA) at 37°C in a humidified incubator with 5% CO_2_.

### 2.3. Immunohistochemistry

HCC tissue specimens were paraffin-embedded and sectioned. Following dewaxing, sections were rehydrated in a descending ethanol gradient. Subsequently, antigen retrieval was performed by heating the slides at 95°C in sodium citrate buffer (pH 6.0) for 30 min. After naturally cooling, the sections were blocked with 3% H_2_O_2_ in methanol for 20 min for inactivation of the endogenous peroxidase. Then, the sections were blocked in 10% goat serum at room temperature for 1 h to reduce nonspecific reactions followed by incubation with anti-NEIL3 antibody (1:50, Thermo Fisher, Waltham, MA, USA) overnight at 4°C. Following incubation with anti-NEIL3 antibodies, the sections were incubated with secondary antibodies. Finally, the sections were stained with diaminobenzidine, counterstained with hematoxylin, and mounted with neutral resin [16, 17].

For the assessment of NEIL3, a semiquantitative histochemistry score (H-score) system was adopted and based on the percentage of positively staining cells (0, 0% positive cells; 1, 1–25% positive cells; 2, 26–50% positive cells; 3, 51–75% positive cells; and 4, 76–100% positive cells) and the staining intensity (0, negative; 1, weak; 2, intermediate; and 3, strong). The final expression score was generated by multiplying the scores of the percentage of positive cells and staining intensity, which ranged from 0 to 12. Scores >6 were regarded as high NEIL3 expression, and scores ≤6 were regarded as low expression [16–18]. The assessment of each section was examined by two independent pathologists, who were blinded to outcomes. If different scores occurred, the slides were reviewed by the two pathologists at a multiheaded microscope until consensus was achieved. Duplicate spots for each tumor showed a good level of homogeneity for the percentage of cells stained and intensity of staining. If there were differences between duplicate tissue cores, the higher score was taken as the final score.

### 2.4. Western Blotting

Total protein was obtained by lysing cells with RIPA buffer. Then, protein samples were subjected to SDS-PAGE and transferred onto PVDF membranes (Millipore, Bedford, MA, USA). After blocking with 5% nonfat milk for 1 h, the membranes were incubated with primary antibody (anti-GADPH, 1:1000, Cell Signaling Technology, Danvers, MA, USA; anti-NEIL3, 1:500, Thermo Fisher) overnight at 4°C. Subsequently, the membranes were washed and incubated with secondary antibodies at room temperature for 1 h. After washing, the membranes were detected with an ECL detection kit (Millipore) [3, 19].

### 2.5. RNA Isolation and Quantitative Real-Time Polymerase Chain Reaction (qRT-PCR)

Total RNA was extracted from tissues and cells with TRIzol reagent (Invitrogen, Carlsbad, CA, USA). Reverse transcription of mRNA was conducted with the Reverse Transcription Kit (TaKaRa, Tokyo, Japan) according to the manufacturer's instructions. QRT-PCR was performed using an SYBR Green master mix (Toyobo, Osaka, Japan), using an ABI 7500 system (Applied Biosystems, Foster City, CA, USA) [20]. GAPDH was used as an internal control. The primer sequences for PCR were as follows: human NEIL3: forward, 5′-TCTCCTGTTTTGGAAGTGCAG-3′, and reverse, 5′-CATTAGCACATCACCTAGCATCC-3′, and human GADPH: forward, 5′-AACAGCCTCAAGATCATCAG-3′, and reverse, 5′-AGTCCTTCCACGATACCAA-3′. Each sample was analyzed in triplicate.

### 2.6. Statistical Analysis

We used SPSS 22 (IBM, Armonk, NY, USA), GraphPad Prism 7.0 (GraphPad Software, Inc., San Diego, CA, USA), and R software (R 3.5.3) for statistical analyses. The Chi-square test and Student's *t*-test were used for evaluating differences between the two categories. For comparisons of survival times, the Kaplan–Meier method was used. The Cox proportional hazard model was employed to identify the prognostic varieties in predicting overall survival (OS) and disease-free survival (DFS). Nomograms models were established by R software (rms package) [17]. Calibration curves were applied to evaluating the performance features of the predictive nomogram. The concordance index (C-index) was applied to assess the forecast precision of the constructed nomogram [17]. *P* < 0.05 was considered statistically significant.

## 3. Results

### 3.1. NEIL3 Expression Is Upregulated in HCC

**Figure 1:**
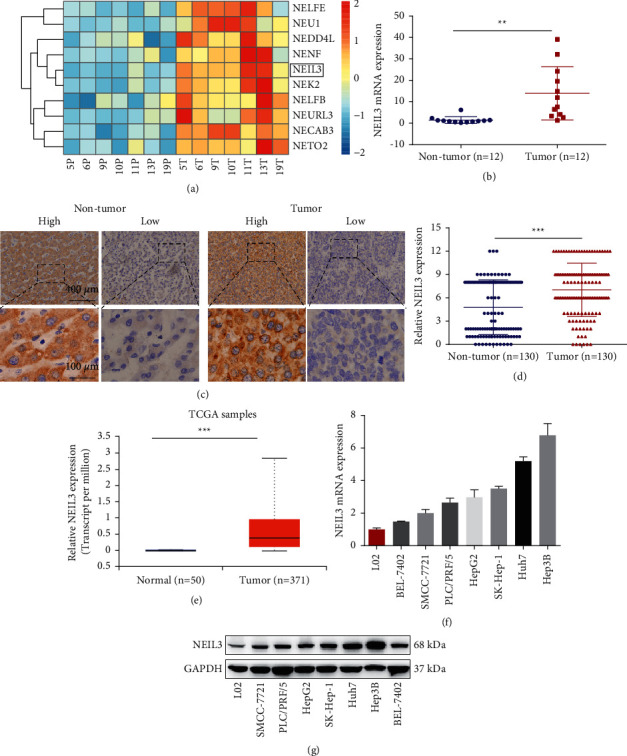
Overexpression of NEIL3 in HCC tissues. (a) A heatmap showed differential expression of genes in seven pair of HCC by mRNA microarray detection. (b) qRT-PCR revealed that the expression of NEIL3 was significantly higher in tumor tissues than that in corresponding adjacent tissues (*n* = 12). (c) NEIL3 protein level was detected in 130 pairs of specimens by immunohistochemistry. (d) Histopathological score of immunohistochemistry showed that NEIL3 protein levels were remarkably upregulated in tumor tissues compared with nontumor tissues. (e) Data from the Cancer Genome Atlas database revealed that the mRNA expression level of NEIL3 was significantly upregulated in HCC compared with that in normal tissues (adapted from UALCAN (http://ualcan.path.uab.edu/index.html)). (f) qRT-PCR revealed that NEIL3 mRNA expression was significantly upregulated in seven HCC cell lines compared with the normal hepatic cell line L02. (g) Western blotting revealed that the NEIL3 protein level was significantly upregulated in seven HCC cell lines compared with the normal hepatic cell line L02. NEIL3, Nei-like DNA glycosylase 3; HCC, hepatocellular carcinoma; qRT-PCR, quantitative real-time polymerase chain reaction. ^*∗∗*^*P* < 0.01 and ^*∗∗∗*^*P* < 0.001.

By analyzing the expression of NEIL3 in our microarray data sets (GSE101728), we found NEIL3 overexpression in HCC tissues (fold change = 23.81; *P* < 0.001; Figure 1(a)). Subsequently, we examined mRNA levels in 12 pairs of HCC samples. The mRNA expression of NEIL3 was significantly higher in tumor tissues than that in the corresponding adjacent tissues (Figure 1(b); fold change = 9.73; *P* = 0.002). Then we expanded the sample size and detected NEIL3 protein levels in 130 pairs of specimens by IHC (Figure 1(c)); the 130 patients consisted of 102 men (78.5%) and 28 women (21.5%) with a median age of 53.0 (range 19–89 years). The histopathological scores revealed that NEIL3 protein expression was remarkably upregulated in tumor tissues compared with nontumor tissues (Figure 1(d); *P* < 0.001). We also determined NEIL3 expression in the Cancer Genome Atlas (TCGA) database. The results showed that the mRNA expression level of NEIL3 was significantly upregulated in HCC tissues compared with that of normal tissues (Figure 1(e); *P* < 0.001).

Finally, we performed qRT-PCR and western blotting to detect NEIL3 mRNA and protein expression in cell lines, respectively. The results showed that NEIL3 mRNA expression was significantly upregulated in seven HCC cell lines compared with the normal hepatic cell line L02 (Figure 1(f)). Consistent with the PCR results, NEIL3 protein was overexpressed in HCC cell lines (Figure 1(g)).

### 3.2. Association between NEIL3 Expression and Clinicopathological Features

To investigate correlations between NEIL3 expression and clinicopathological features in patients with HCC, we divided the patients into high and low NEIL3 expression groups based on the histopathological score. A chi-square test revealed that high NEIL3 expression was significantly correlated with BCLC stage (*P*=0.004) and TNM stage (*P*=0.005; Table 1). However, no significant statistical associations were observed between NEIL3 expression and clinicopathological parameters, such as gender, age, hepatitis B surface antigen (HBsAg) positivity, cirrhosis, preoperative serum *α*-fetoprotein (AFP), tumor size, tumor number, or tumor differentiation, in either cohort (Table 1).

### 3.3. Association between NEIL3 Expression and Survival Time

**Figure 2:**
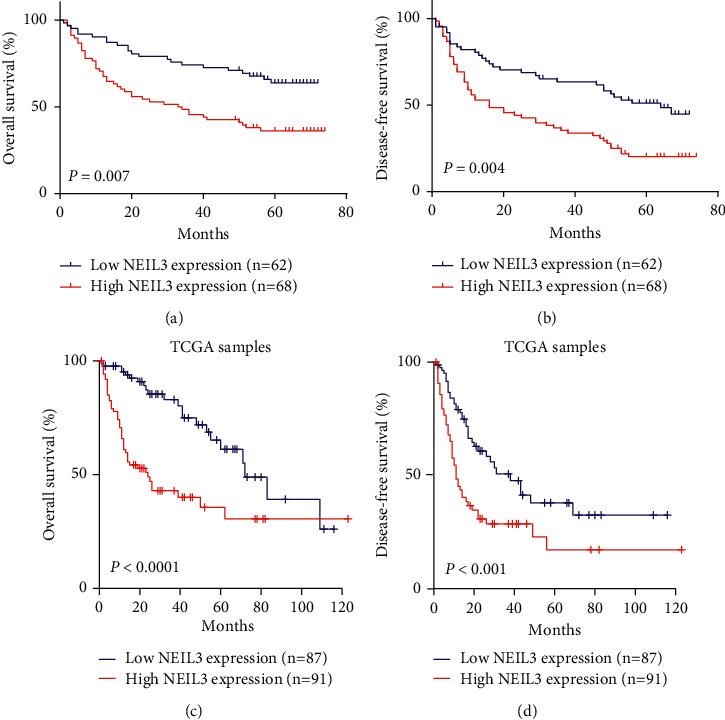
Kaplan–Meier survival curves according to NEIL3 expression in HCC patients. (a, c) Data from our hospital cohort and the Cancer Genome Atlas database revealed that overexpression of NEIL3 was significantly correlated with poor OS. (b, d) Data from our hospital cohort and the Cancer Genome Atlas database revealed that overexpression of NEIL3 was significantly correlated with poor DFS. Figures (c) and (d) were adapted from GEPIA (http://gepia.cancer-pku.cn/). NEIL3, Nei-like DNA glycosylase; HCC, hepatocellular carcinoma; OS, overall survival; DFS, disease-free survival.

In general, the range of follow-up of the 130 HCC patients was 1.4–72.0 months; 1-, 3-, and 5-year OS rates were 78.5%, 59.2%, and 36.9%, respectively, while 1-, 3-, and 5-year DFS rates were 65.4%, 46.2%, and 27.7%, respectively. To determine the association between NEIL3 expression and prognosis, Kaplan–Meier survival curves and log-rank test were applied to analyze OS and DFS in the high and low NEIL3 expression groups. As shown in Figures 2(a) and 2(b), OS and DFS rates in the high NEIL3 expression group were significantly poorer than those in the low NEIL3 expression group (Figure 2(a), *P*=0.007; Figure 2(b), *P*=0.004). Specifically, 1-, 3- and 5-year OS rates in the high NEIL3 expression group were 67.6%, 45.6%, and 26.5%, respectively. In the low NEIL3 expression group, 1-, 3-, and 5-year OS rates were 90.3%, 74.2%, and 48.4%, respectively. In the high NEIL3 expression group, 1-, 3-, and 5-year DFS rates were 52.9%, 35.3%, and 14.7%, respectively, while in the low NEIL3 expression group, 1-, 3-, and 5-year DFS rates were 79.0%, 58.1%, and 41.9%, respectively. These results were further confirmed by the TCGA database, which indicated that HCC patients with higher mRNA expression of NEIL3 had worse OS and DFS (Figure 2(c), *P* < 0.0001; Figure 2(d), *P* < 0.001).

### 3.4. Correlation of NEIL3 Expression with Prognosis in Subgroups

**Figure 3:**
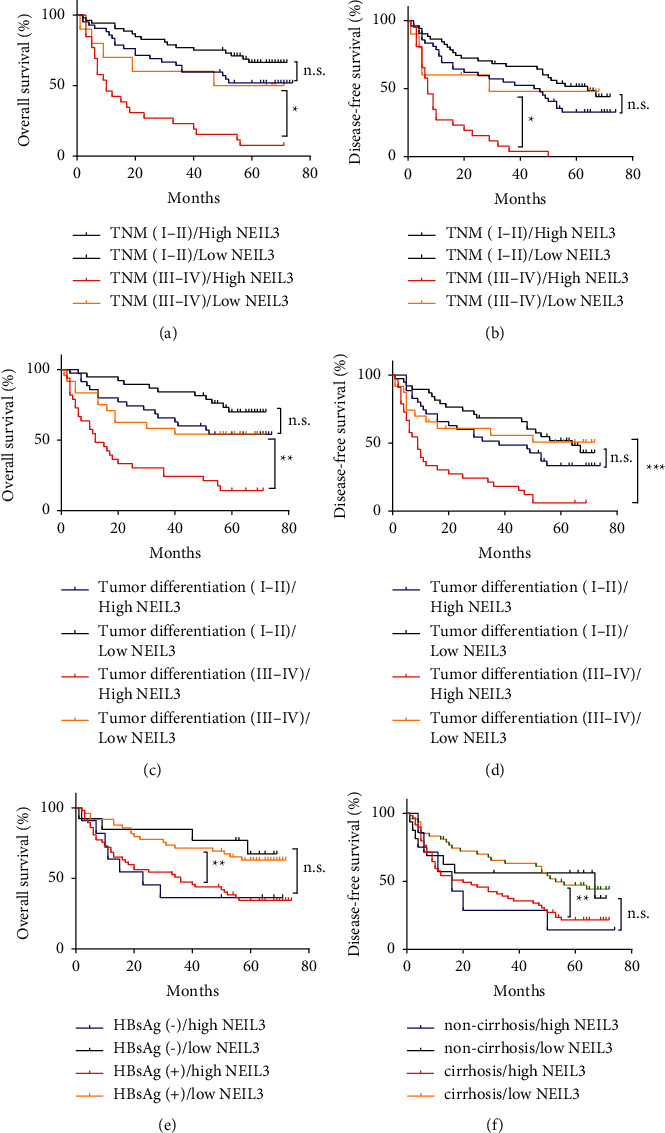
Kaplan–Meier survival curves according to NEIL3 expression in HCC patients stratified by TNM stage, tumor differentiation, HBsAg expression, or cirrhosis. (a, b) Advanced TNM stage patients with high NEIL3 expression had a worse OS (*P*=0.019) and DFS (*P*=0.01) compared with advanced TNM stage patients with low NEIL3 expression. (c, d) Poor tumor differentiation patients with high NEIL3 expression had a worse OS (*P*=0.003) and DFS (*P* < 0.001) compared with the poor tumor differentiation patients with low NEIL3 expression. (e) HBsAg positive patients with high NEIL3 expression had a worse OS (*P*=0.002) compared with HBsAg positive patients with low NEIL3 expression. (f) cirrhosis patients with high NEIL3 expression had a worse DFS (*P*=0.004) compared with cirrhosis positive patients with low NEIL3 expression. NEIL3, Nei-like DNA glycosylase; HCC, hepatocellular carcinoma; OS, overall survival; DFS, disease-free survival.

As some clinical features are known to be prognostic factors for HCC, we divided our HCC cohort into several subgroups to analyze the prognostic role of NEIL3. We found that for patients with high TNM stage, high NEIL3 expression indicated worse OS (*P*=0.019; Figure 3(a)) and DFS (*P*=0.01; Figure 3(b)) compared with high TNM stage patients with low NEIL3 expression. However, there was no remarkable difference between low TNM stage patients with high and low NEIL3 expression in OS (*P*=0.101; Figure 3(a)) and DFS (*P*=0.114; Figure 3(b)). Similarly, for patients with poor tumor differentiation, high NEIL3 expression indicated worse OS (*P*=0.003; Figure 3(c)) and DFS (*P* < 0.001; Figure 3(d)) compared with the patients with low NEIL3 expression. However, there was no remarkable difference between well tumor differentiation patients with high and low NEIL3 expression in OS (*P*=0.168; Figure 3(c)) and DFS (*P*=0.044; Figure 3(c)). Moreover, we found that HBsAg positive patients with high NEIL3 expression had worse OS (*P*=0.002; Figure 3(e)) compared with HBsAg positive patients with low NEIL3 expression, and cirrhosis patients with high NEIL3 expression had worse DFS (*P*=0.004; Figure 3(f)) compared with cirrhosis patients with low NEIL3 expression. These results indicated that for HCC patients with advanced TNM stage, poorly differentiated tumor, HBsAg positive, or cirrhosis, NEIL3 expression might serve as a prognostic factor.

### 3.5. Univariate and Multivariate Analyses of Predictive Factors Associated with OS and DFS in HCC Patients

The relationship between all clinicopathological parameters and prognosis was further analyzed by univariate and multivariate analyses. For OS, the univariate analysis indicated that the significant factors that influenced OS rate were NEIL3 expression (hazard ratio (HR) 2.368; 95% confidence interval (CI) 1.414–3.968; *P*=0.001), TNM stage (HR 3.257; 95% CI 1.982–5.352; *P* < 0.001), AFP (HR 2.550; 95% CI 1.410–4.614; *P*=0.002), tumor size (HR 2.571; 95% CI 1.531–4.312; *P* < 0.001), tumor differentiation (HR 2.710; 95% CI 1.649–4.451; *P* < 0.001), and BCLC stage (HR 2.012; 95% CI 1.231–3.286; *P*=0.005). Multivariate analysis using the five mentioned factors revealed that NEIL3 expression (HR 1.980; 95% CI 1.159–3.380; *P*=0.012), TNM stage (HR 10.529; 95% CI 1.399–79.261; *P*=0.022), AFP (HR 1.967; 95% CI 1.073–3.605; *P*=0.029), tumor size (HR 2.171; 95% CI 1.242–3.796; *P*=0.007), and tumor differentiation (HR 2.032; 95% CI 1.223–3.376; *P*=0.006) serve as independent prognostic factors for OS (Table 2). For DFS, univariate analysis showed that NEIL3 expression (HR 2.172; 95% CI 1.392–2.390; *P* = 0.001), TNM stage (HR 2.873; 95% CI 1.826–4.520; *P* < 0.001), tumor differentiation (HR 1.970; 95% CI 1.284–3.022; *P*=0.002), tumor size (HR 1.745; 95% CI 1.135–2.683; *P*=0.011), AFP (HR 1.864; 95% CI 1.160–2.995; *P*=0.010), and BCLC stage (HR 1.948; 95% CI 1.263–3.004; *P*=0.003) were significant variables. These factors were then selected for DFS multivariate analysis, which revealed that NEIL3 expression (HR 1.947; 95% CI 1.233–3.074; *P*=0.004), TNM stage (HR 3.131; 95% CI 1.165–8.416; *P*=0.024), and tumor differentiation (HR 1.691; 95% CI 1.089–2.625; *P*=0.019) were significant factors of DFS (Table 3). In summary, the multivariate Cox proportional hazards model indicated that NEIL3 expression in HCC serves as an independent prognostic factor for OS and DFS.

### 3.6. Validation of the Prognostic Value of NEIL3 in HCC Based on Nomograms

**Figure 4:**
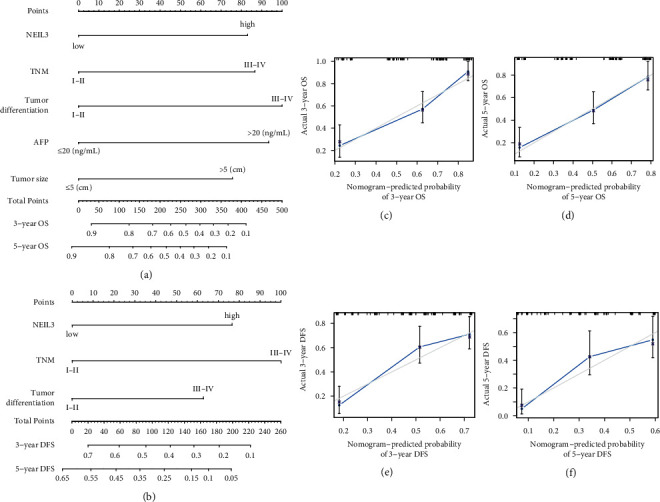
Prognostic nomogram generation for predicting OS and DFS in patients with HCC. Nomogram-predicted OS (a) and DFS (b) for HCC patients. Calibration curves for predicting OS at 3 years (c) and 5 years (d). Calibration curves for predicting DFS at 3 years (e) and 5 years (f). Actual OS or DFS are plotted on the *y*-axis; nomogram-predicted probabilities of OS or DFS are plotted on the *x*-axis. HCC, hepatocellular carcinoma; OS, overall survival; DFS, disease-free survival.

We further established two nomograms for OS and DFS, respectively, based on tumor NEIL3 level and other independent prognostic factors, which were identified via multivariate analyses (Figures 4(a) and 4(b)). To validate the accuracy of the two prognostic nomograms, the calibration curves were conducted. As shown in calibration curves (Figures 4(c)–4(f)), there were excellent agreements between the prediction by nomogram and the actual observed outcome (3- and 5-year OS and DFS) after surgery. The C-index (Harrell's concordance index) of NEIL3-based nomogram was 0.749 (95% CI 0.697–0.801) for the forecast of OS, which was superior to that of the TNM staging system alone (C-index: 0.633 (95% CI 0.577–0.689)). Likewise, the C-index for DFS was 0.678 (95% CI 0.624–0.732), which was also superior to that of the TNM staging system (C-index: 0.619 (95% CI 0.571–0.667)). These findings showed that the NEIL3-based nomogram is a promising prognostic model for HCC patients after curative hepatectomy.

## 4. Discussion

Over the past decades, accumulating evidence has suggested that NEIL3 expression is associated with human tumors [13, 21–23]. However, few studies have investigated the role of NEIL3 in HCC. In the present study, we revealed that NEIL3 expression is significantly upregulated in tumor tissues and HCC cell lines. Kaplan–Meier survival curves revealed that NEIL3 overexpression is significantly associated with worse OS and DFS rates. Moreover, univariate and multivariate analyses demonstrated that tumor NEIL3 serves as an independent prognostic factor for OS and DFS, and the nomogram further validated the prognostic value.

As for the potential mechanism of NEIL3 in the onset and progression of the tumor, previous studies have indicated that it may be associated with the dysfunction of telomere [7, 24]. Telomeres are located in the ends of mammalian chromosomes, which are susceptible to reactive oxygen and then result in telomere erosion, DNA bridging, and chromosomal instability [25, 26]. At present, the dysfunction has also been shown to result in the initiation and progression of tumor in the colon, prostate, and breast [27–29]. A previous study revealed that most oxidative lesions are restored by the base excision repair pathway [30], which is initiated by a DNA glycosylase. NEIL3 is a member of the Nei-like DNA glycosylase family and preferentially removes oxidative lesions from telomere DNA [7]. However, in pathological conditions, the abnormally expressed NEIL3 overprotects the integrity of telomeric sequences, which allows cells to proliferate unrestrictedly, leading to the onset of tumors. These findings may partially explain why high tumor NEIL3 expression is correlated with poor outcome in patients with HCC, but its exact mechanism needs to be further explored.

At present, hepatectomy and liver transplantation are major radical treatments for HCC [31]. However, most patients are diagnosed at advanced stages due to atypical symptoms and a lack of early diagnosis indexes, which results in less effective surgery [2, 31, 32]. Promising prognostic indicators will be significant for the timely adjustment of postoperative treatment regimens. In the present study, Kaplan–Meier analyses revealed that elevated NEIL3 level is correlated with poor OS and DFS. In the subgroup analysis, we found that for HCC patients with advanced TNM stage, poorly differentiated tumor, HBsAg positive or cirrhosis, and NEIL3 expression could serve as a prognostic factor. The results revealed the prognostic value of NEIL3. However, the mechanism behind this phenomenon still needs to be further investigated. Nomograms are widely used as prognostic models in medicine, which have been shown to be with higher prognostic accuracy than the TNM staging system for the majority of cancer types [17, 33]. In the present study, we established NEIL3-based nomograms to more accurately predict the long-term outcomes of postoperative patients by integrating diverse prognostic variables based on multivariate analyses. It demonstrated that NEIL3-based nomograms could be a more precise predictive model for HCC patients. The above results suggested that NEIL3 could serve as a promising prognostic biomarker for HCC patients.

Additionally, it has been reported that DNA damage repair determines the response to chemotherapeutics (such as 5-fluorouracil, capecitabine, and so on) in some tumors including HCC [34, 35]. A recent study demonstrated that deficiency of NEIL3 contributes to chemoresistance through modulating DNA damage repair in prostate cancer [36], suggesting the potential clinical and therapeutic application of NEIL3. Our study found that NEIL3 was upregulated in HCC and associated with poor prognosis of patients. Further study is needed to evaluate whether NEIL3 expression can be a prognostic marker for systemic chemotherapy.

## 5. Conclusions

The present study indicated that NEIL3 expression is significantly upregulated in HCC. The tumor NEIL3 level could serve as a promising prognostic indicator for OS and DFS rates in HCC patients after curative hepatectomy. In the future, further investigation will be performed to delineate the mechanism of how NEIL3 contributes to the pathophysiology of HCC.

## Figures and Tables

**Table 1:** Association of NEIL3 level with clinicopathological parameters of HCC patients.

Parameters	All patients	NEIL3 expression		*χ* ^2^	*P* value
	(*n* = 130)	High (*n* = 68)	Low (*n* = 62)		
Gender				0.494	0.482
Male	102	55	47		
Female	28	13	15		
Age (years)				0.226	0.634
≤55	79	40	39		
>55	51	28	23		
HBsAg				1.379	0.240
Negative	22	9	13		
Positive	108	59	49		
Cirrhosis				1.336	0.248
Negative	24	10	14		
Positive	106	58	48		
AFP (ng/mL)				1.706	0.192
≤20	45	20	25		
>20	85	48	37		
Tumor size (cm)				2.473	0.116
≤5	64	29	35		
>5	66	39	27		
Tumor number				0.001	0.991
Solitary	88	46	42		
Multiple	42	22	20		
Tumor differentiation				1.270	0.260
I‐II	73	35	38		
III‐IV	57	33	24		
TNM stage				7.915	**0.005**
I‐II	94	42	52		
III‐IV	36	26	10		
BCLC stage					
0-A	84	36	48	8.499	**0.004**
B-C	46	32	14		

NEIL3, Nei-like DNA glycosylase 3; HCC, hepatocellular carcinoma; HBsAg, hepatitis B surface antigen; AFP, serum alpha-fetoprotein; TNM, tumor-nodes-metastasis; BCLC, Barcelona Clinic Liver Cancer. Bold values represent *P* < 0.05, which is statistically significant.

**Table 2:** Univariate and multivariate analyses of prognostic factors of OS.

Factors	Univariate analysis		Multivariate analysis	
	HR (95% CI)	*P* value	HR (95% CI)	*P* value
NEIL3 (high vs. low)	2.368 (1.414–3.968)	**0.001**	1.980 (1.159–3.380)	**0.012**
TNM (III–IV vs. I–II)	3.257 (1.982–5.352)	**<0.001**	10.529 (1.399–79.261)	**0.022**
AFP (ng/mL) (>20 vs. ≤20)	2.550(1.410–4.614)	**0.002**	1.967 (1.073–3.605)	**0.029**
Tumor size (cm) (>5 vs. ≤5)	2.571 (1.531–4.312)	**<0.001**	2.171 (1.242–3.796)	**0.007**
Tumor differentiation (III–IV vs. I–II)	2.710 (1.649–4.451)	**<0.001**	2.032 (1.223–3.376)	**0.006**
BCLC (0-A vs. B-C)	2.012 (1.231–3.286)	**0.005**	0.164 (0.022–1.233)	0.079
Tumor number (multiple vs. solitary)	1.032 (0.614–1.736)	0.905	—	n.a.
Gender (male vs. female)	0.890 (0.500–1.584)	0.691	—	n.a.
Age, years (≥55 vs. <55)	0.803 (0.474–1.360)	0.415	—	n.a.
HBsAg (positive vs. negative)	1.115 (0.583–2.133)	0.742	—	n.a.
Cirrhosis (positive vs. negative)	0.897 (0.479–1.679)	0.734	—	n.a.

NEIL3, Nei-like DNA glycosylase 3; HCC, hepatocellular carcinoma; HR, hazard ratio; CI, confidence interval; TNM, tumor-nodes-metastasis;; BCLC, Barcelona Clinic Liver Cancer; AFP, serum alpha-fetoprotein; HBsAg, hepatitis B surface antigen; n.a., not applicable. Bold values represent *P* < 0.05, which is statistically significant.

**Table 3:** Univariate and multivariate analyses of prognostic factors of DFS.

Factors	Univariate analysis		Multivariate analysis	
	HR (95% CI)	*P* value	HR (95% CI)	*P* value
NEIL3 (high vs. low)	2.172 (1.392–2.390)	**0.001**	1.947 (1.233–3.074)	**0.004**
TNM (III–IV vs. I–II)	2.873 (1.826–4.520)	**<0.001**	3.131 (1.165–8.416)	**0.024**
Tumor differentiation (III–IV vs. I–II)	1.970 (1.284–3.022)	**0.002**	1.691 (1.089–2.625)	**0.019**
Tumor size, cm (>5 vs. ≤5)	1.745 (1.135–2.683)	**0.011**	1.403 (0.866–2.273)	0.169
AFP, ng/mL (>20 vs. ≤20)	1.864 (1.160–2.995)	**0.010**	1.443 (0.887–2.349)	0.140
BCLC (0-A vs. B-C)	1.948 (1.263–3.004)	**0.003**	0.628 (0.236–1.669)	0.350
Tumor number (multiple vs. solitary)	1.131 (0.719–1.779)	0.596	—	n.a.
Gender (male vs. female)	1.043 (0.620–1.756)	0.874	—	n.a.
Age, years (≥55 vs. <55)	0.888 (0.564–1.396)	0.606	—	n.a.
HBsAg (positive vs. negative)	1.044 (0.597–1.823)	0.881	—	n.a.
Cirrhosis (positive vs. negative)	1.045 (0.589–1.854)	0.881	—	n.a.

NEIL3, Nei-like DNA glycosylase 3; HCC, hepatocellular carcinoma; HR, hazard ratio; CI, confidence interval; TNM, tumor-nodes-metastasis; BCLC, Barcelona Clinic Liver Cancer; AFP, serum alpha-fetoprotein; HBsAg, hepatitis B surface antigen; n.a., not applicable.

## Data Availability

The data used to support the findings of this study are available from the corresponding author upon request.
